# A Robust Seven-Gene Signature Associated With Tumor Microenvironment to Predict Survival Outcomes of Patients With Stage III–IV Lung Adenocarcinoma

**DOI:** 10.3389/fgene.2021.684281

**Published:** 2021-09-06

**Authors:** Hao Zhao, Xuening Zhang, Lan Guo, Songhe Shi, Ciyong Lu

**Affiliations:** ^1^Department of Medical Statistics and Epidemiology, School of Public Health, Sun Yat-sen University, Guangzhou, China; ^2^Department of Epidemiology and Biostatistics, College of Public Health, Shandong University, Jinan, China; ^3^Department of Epidemiology and Biostatistics, College of Public Health, Zhengzhou University, Zhengzhou, China

**Keywords:** tumor microenvironment, immune, lung adenocarcinoma, stromal, TCGA

## Abstract

**Background:**

Due to the relatively insidious early symptoms of lung adenocarcinoma (LUAD), most LUAD patients are at an advanced stage at the time of diagnosis and lose the best chance of surgical resection. Mounting evidence suggested that the tumor microenvironment (TME) was highly correlated with tumor occurrence, progress, and prognosis. However, TME in advanced LUAD remained to be studied and reliable prognostic signatures based on TME in advanced LUAD also had not been well-established. This study aimed to understand the cell composition and function of TME and construct a gene signature associated with TME in advanced LUAD.

**Methods:**

The immune, stromal, and ESTIMATE scores of each sample from The Cancer Genome Atlas (TCGA) database were, respectively, calculated using an ESTIMATE algorithm. The LASSO and Cox regression model were applied to select prognostic genes and to construct a gene signature associated with TME. Two independent datasets from the Gene Expression Omnibus (GEO) were used for external validation. Twenty-two subsets of tumor-infiltrating immune cells (Tiics) were analyzed using the CIBERSORT algorithm.

**Results:**

Favorable overall survival (OS) and progression-free survival (PFS) were found in patients with high immune score (*p* = 0.048 and *p* = 0.028; respectively) and stromal score (*p* = 0.024 and *p* = 0.025; respectively). Based on the immune and stromal scores, 453 differentially expressed genes (DEGs) were identified. Using the LASSO and Cox regression model, a seven-gene signature containing AFAP1L2, CAMK1D, LOXL2, PIK3CG, PLEKHG1, RARRES2, and SPP1 was identified to construct a risk stratification model. The OS and PFS of the high-risk group were significantly worse than that of the low-risk group (*p* < 0.001 and *p* < 0.001; respectively). The receiver operating characteristic (ROC) curve analysis confirmed the good potency of the seven-gene signature. Similar findings were validated in two independent cohorts. In addition, the proportion of macrophages M2 and Tregs was higher in high-risk patients (*p* = 0.041 and *p* = 0.022, respectively).

**Conclusion:**

Our study established and validated a seven-gene signature associated with TME, which might serve as a prognosis stratification tool to predict survival outcomes of advanced LUAD patients. In addition, macrophages M2 polarization may lead to worse prognosis in patients with advanced LUAD.

## Introduction

Lung cancer ranks first in the incidence and mortality of all malignant tumors worldwide (Bray et al., 2018). The 5-year survival rate of lung cancer patients is less than 20% (Herbst et al., 2018). Lung adenocarcinoma (LUAD) is the most common histological subtype of non-small cell lung cancer (NSCLC), which accounts for about 40% of all lung malignancies and usually occurs in the outer area of the lung (Chen et al., 2014). Clinical studies have shown that nearly 70% of LUAD patients are discovered in stage III–IV, and 57% of LUAD patients have already developed distant metastasis at the time of initial diagnosis, and have lost the best opportunity for surgical resection (Jemal et al., 2017).

In recent years, significant progress has been made in the research of molecular genetics and immunotherapy of lung cancer, and molecular typing based on genetic characteristics has brought the treatment of advanced lung cancer into the era of personalized molecular targeted therapy (Subramanian and Govindan, 2008). EGFR-Inhibitor and BRAF(V600E)-mutant, rearrangements of ALK or ROS1 genes, as well as immune checkpoint inhibitor antibodies against PD-1 or PD-L1 have been approved for the treatment of advanced LUAD (Vargas and Harris, 2016; Hirsch et al., 2017). At present, the TNM staging system is still the most effective tool to judge the survival of patients and guide clinical treatment strategies, but the evaluation effect for advanced survival is not good (Goldstraw et al., 2016). Therefore, looking for a new survival predictor for advanced LUAD patients is particularly important for personalized treatment of clinical decision-making and prognostic health management.

Tumor microenvironment (TME) refers to the surrounding microenvironment of tumor cells, including immune cells, stromal cells, endothelial cells, inflammatory cells, and fibroblasts (Neal et al., 2018). Among them, immune cells and stromal cells are two major non-tumor cell components, which have been considered important for the diagnosis and prognostic evaluation of cancer patients (Gajewski et al., 2013). Therefore, understanding the cell composition and function of TME will bring a new dawn to patients with advanced LUAD in terms of immunity and targeted therapy and improvement of prognosis.

The continuous improvement and development of public databases, such as The Cancer Genome Atlas (TCGA) database and Gene Expression Omnibus (GEO) database, provide reliable data resources for TME research (Cancer Genome Atlas Research Network, Weinstein et al., 2013; NCBI Resource Coordinators, 2016). Yoshihara et al. (2013) first proposed the ESTIMATE algorithm in 2013. This algorithm uses the unique properties of the transcription profile of cancer samples to infer infiltrating stromal/immune cells. According to reports, researchers have explored the tumor characteristics and prognosis assessment of liver cancer (Li et al., 2019), breast cancer (Bai et al., 2019), and clear cell renal cell carcinoma (Luo et al., 2020) based on the ESTIMATE algorithm. However, the value of immune and stromal scores for advanced LUAD has not been verified.

In the present study, the immune and stromal scores were estimated using the ESTIMATE algorithm based on the transcription profile of LUAD patients with stage III–IV. A robust gene signature based on immune-stromal score was subsequently developed for prognosis stratification in advanced LUAD. Finally, we explored the relationship between high-/low-risk advanced LUAD patients and immune cell infiltration based on the CIBERSORT method, so as to provide some references for combined immunotherapy and targeted therapy for advanced LUAD patients.

## Materials and Methods

### Data Collection and Processing

We obtained the fragments per kilobase per million (FPKM) data of RNA-Seq from the TCGA-LUAD cohort^1^, including 535 LUAD patients and 59 normal samples. Next, the FPKM data were transferred to transcripts per million (TPM) expression data. The gene expression levels of duplicate samples were averaged, and normal samples were deleted for subsequent analysis.

We used the GDC tool and cBioPortal website^2^ to download the corresponding clinical information, including age, gender, history of smoking, tumor laterality, metastasis, lymph node status, pathological T stage, stage, and prognostic information. In this study, only patients with stage III–IV were included and patients with incomplete key clinical information were excluded. Finally, a total of 103 advanced LUAD patients were included for follow-up analysis. We utilized the “limma” package for normalization processing, and then immune, stromal, and ESTIMATE scores were calculated using ESTIMATE algorithm. Two independent datasets from the GEO database^3^, namely, 27 LUAD patients with stage III–IV from Series GSE81089 and 53 LUAD patients with stage III–IV from Series GSE41271, were used for external validation in this study. For all patients from the GEO database, a normalized expression matrix was used for subsequent analyses.

### Correlations Between Prognoses and Immune/Stromal Scores

Overall survival (OS) was used as the primary prognosis endpoint, and progression-free survival (PFS) was used as the secondary prognosis endpoint. According to the stromal and immune scores of each advanced LUAD patient, the best cutoff value based on the R package “maxstat” (i.e., the maximum selective rank statistic method) (Ritchie et al., 2015) was used to divide the patients into high-score and low-score groups. Based on “survival” packages, the Kaplan–Meier (K–M) survival curve analysis and log-rank tests were used to compare the prognoses of the two groups.

### Differentially Expressed Gene (DEG) Screening

The “limma” package in R software was used to screen for DEGs between high-score and low-score groups of immune score and stromal score. In this study, an adjusted *p*-value < 0.05 and fold change ≥1.5 were regarded as the critical value for screening DEGs. The immune-related DEGs and stromal-related DEGs showing the same expression trend were selected for further analysis using a Venn diagram. We used the “pheatmap” package to generate the immune-related heatmap and stromal-related heatmap.

### DEG Functional Enrichment Analysis

The David online database^4^ was used to explore the potential functions of DEGs. Gene ontology (GO) analysis included biological processes (BP), molecular functions (MF), and cellular components (CC), which are demonstrated by a bar plot. Kyoto Encyclopedia of Genes and Genomes (KEGG) analysis was performed to conduct the pathway analysis, which was illustrated by a dot plot. With false discovery rate (FDR) < 0.05 as cutoff value, all enrichment results were visualized with “ggplot2” package.

### Construction of Gene Signature and Survival Analysis

Firstly, the univariate Cox model was used to determine the relationship between TME-related DEG expression and patient’s survival. Then, the least absolute shrinkage and selection operator (LASSO) regression analysis was used to further screen out key genes from significant DEGs in the univariate analysis. LASSO regression increases penalty function on the basis of the least squares method, which can reduce the number of variables and avoid overfitting effectively (Bovelstad et al., 2007). Finally, the key genes screened by LASSO were included in multivariate Cox analysis, and the gene signature (risk score) formula was constructed according to the analysis results.

The risk score was calculated as follows: risk score = ∑ (βi ^∗^ Expi) (“i” = the number of prognostic hub genes, “βi” represents the coefficient of each gene, and “Expi” represents gene expression).

In addition, advanced LAUD patients were divided into high-risk and low-risk groups according to the median risk score. The receiver operating characteristic (ROC) curves and the consistency index (C-index) were then used to assess the predictive ability of the risk score. The K–M curves and log-rank tests were used to analyze the difference in survival between the high-risk group and the low-risk group. Furthermore, the independent prognostic value of the gene signature was explored by multivariate Cox analysis combined with other clinicopathologic characteristics.

### Validation of Gene Signature in the Testing Dataset

The GSE81089 and GSE41271 independent datasets were used for verification. According to the gene signature calculation formula of the training dataset, the samples in the test dataset were divided into the high-risk group and the low-risk groups. The K–M survival analysis and ROC curves were used to evaluate the predictive ability of this model. Immunohistochemistry (IHC) images of the selected prognosis-related genes in tumor and normal tissue were retrieved from the Human Protein Atlas online database^5^.

### Estimating the Composition of Immune Cells

CIBERSORT is a deconvolution algorithm based on the principle of linear support vector regression to describe the infiltration of immune cells in the sample (Shen-Orr et al., 2010). LM22 is composed of 547 genes that accurately distinguish 22 human hematopoietic cell phenotypes, including seven T-cell types, naïve and memory B cells, plasma cells, NK cells, and myeloid subsets (Newman et al., 2015). We used CIBERSORT and LM22 to jointly estimate the scores of 22 human immune cell types in advanced LAUD patients from the TCGA cohort. For each sample, the sum of all estimated immune cell type scores was equal to 1. We compared differences in the composition of immune cell types between high-risk and low-risk groups.

### Statistical Analysis

Statistical analysis was performed using R software (version 3.6.1). All statistical tests were two sided and *p*-value < 0.05 indicated statistical significance.

## Results

### Estimation of Immune Score and Stromal Score

We included 103 LUAD samples from the TCGA database, of which 78 (75.73%) were in stage III and 25 (24.27%) were in stage IV. The clinical and pathological characteristics of the included patients are listed in Table 1. Among them, elderly LUAD patients (≥65 years) accounted for 53.40%, and the proportion of LUAD patients with a history of smoking was as high as 84.47%.

**TABLE 1:** Clinical characteristics of 103 advanced LUAD patients included in the study from the TCGA cohort.

Variables	Total (*N* = 103)	Death		Log-rank *P*	Progress or Death		Log-rank *P*
		No (*N* = 44)	Yes (*N* = 59)		No (*N* = 35)	Yes (*N* = 68)	
Age				0.766			0.378
<65	48 (46.60)	21 (43.75)	27 (56.25)		18 (37.50)	30 (62.50)	
≥65	55 (53.40)	23 (41.82)	32 (58.18)		17 (30.91)	38 (69.09)	
Gender				0.915			0.549
Female	55 (53.40)	24 (43.64)	31 (56.36)		21 (38.18)	34 (61.82)	
Male	48 (46.60)	20 (41.67)	28 (58.33)		14 (29.17)	34 (70.83)	
Smoke				0.148			0.130
No	16 (15.53)	12 (75.00)	4 (25.00)		10 (62.50)	6 (37.50)	
Yes	87 (84.47)	32 (36.78)	55 (63.22)		25 (28.74)	62 (71.26)	
Tumor laterality				0.227			0.493
Left	41 (39.81)	16 (39.02)	25 (60.98)		14 (34.15)	27 (65.85)	
Right	62 (60.19)	28 (45.16)	34 (54.84)		21 (33.87)	41 (66.13)	
Metastasis				0.928			0.820
M0	79 (76.70)	35 (44.30)	44 (55.70)		29 (36.71)	50 (63.29)	
M1	24 (23.30)	9 (37.50)	15 (62.50)		6 (25.00)	18 (75.00)	
Lymph node status				0.098			0.145
No	17 (16.50)	11 (64.71)	6 (35.29)		10 (58.82)	7 (41.18)	
Yes	86 (83.50)	33 (38.37)	53 (61.63)		25 (29.07)	61 (70.93)	
Pathological T stage				0.063			0.228
T1–T2	67 (65.05)	33 (49.25)	34 (50.75)		26 (38.81)	41 (61.19)	
T3–T4	36 (34.95)	11 (30.56)	25 (69.44)		9 (25.00)	27 (75.00)	
Stage				0.950			0.853
III	78 (75.73)	34 (43.59)	44 (56.41)		28 (35.90)	50 (64.10)	
IV	25 (24.27)	10 (40.00)	15 (60.00)		7 (28.00)	18 (72.00)	

The immune, stromal, and ESTIMATE scores of each sample were, respectively, calculated using an ESTIMATE algorithm. The immune score ranged from −941.95 to 2,940.32, the stromal score ranged from −1,755.55 to 1,923.43, and the ESTIMATE score ranged from −2,298.51 to 4,012.25.

### Immune Score and Stromal Score Were Significantly Related to Advanced LUAD Survival Outcomes

**FIGURE 1:**
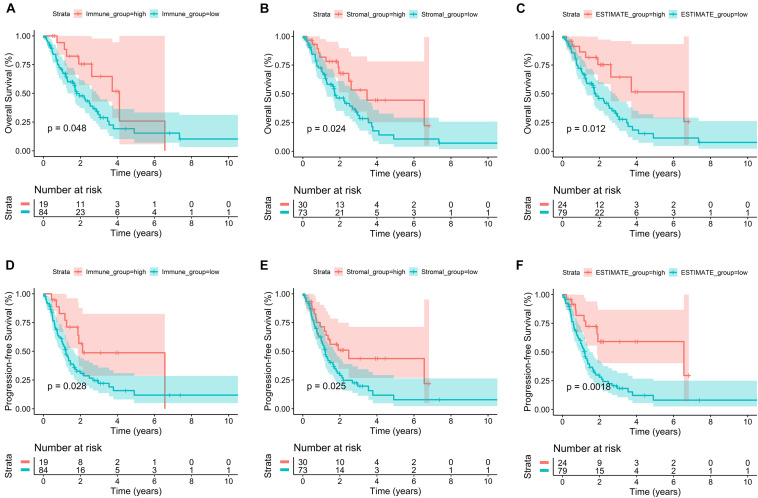
Correlation of immune score and stromal score with advanced LUAD survival outcomes. **(A–C)** K–M survival curves of the relationships between different score levels and OS showed that patients with lower immune, stromal, and ESTIMATE scores had worse OS outcomes (*p* = 0.048, *p* = 0.024, and *p* = 0.012, respectively). **(D–F)** K–M survival curves of the relationships between different score levels and PFS showed that patients with lower immune, stromal, and ESTIMATE scores had worse PFS outcomes (*p* = 0.028, *p* = 0.025, and *p* = 0.002, respectively).

Lung adenocarcinoma samples were divided into high-score and low-score groups, based on the best cutoff value of immune score, stromal score, and ESTIMATE score, respectively. The K–M survival curves were performed to evaluate the relationships between different score levels and survival outcome. K–M survival curves of the relationships between different score levels and OS showed that patients with lower immune, stromal, and ESTIMATE scores had worse OS outcomes (*p* = 0.048, *p* = 0.024, and *p* = 0.012, respectively; Figures 1A–C). Consistently, K–M survival curves of the relationships between different score levels and PFS showed that patients with lower immune, stromal, and ESTIMATE scores had worse PFS outcomes (*p* = 0.028, *p* = 0.025, and *p* = 0.002, respectively; Figures 1D–F). These observations consistently suggested that advanced LUAD patients with a higher immune score or stromal score had a more favorable outcome.

### Identification of DEGs Based on Immune Score and Stromal Score in Advanced LUAD

**FIGURE 2:**
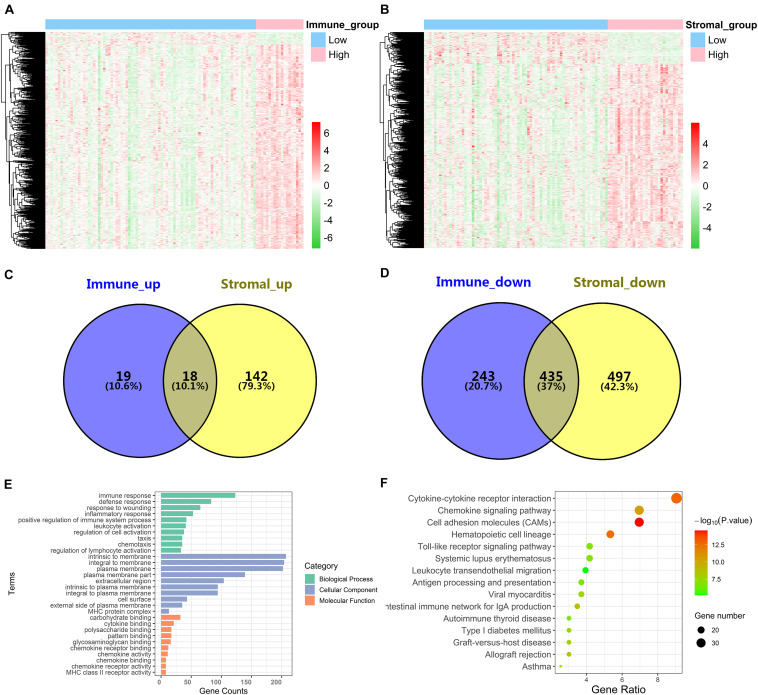
Identification of DEGs and function and pathway enrichment analysis. **(A)** A heatmap of 715 DEGs between patients with high or low immune scores. **(B)** A heatmap of 1,092 DEGs between patients with high or low stromal scores. **(C,D)** Cross-upregulated and cross-downregulated DEGs between the immune and stromal groups. **(E,F)** Function and pathway enrichment analysis of DEGs by GO and KEGG.

In order to explore the DEGs closely related to the TME, the “limma” package was used to process the Affymetrix microarray data from 103 advanced LUAD patients. Figure 2A showed a heatmap of 715 DEGs between high and low immune scores, and Figure 2B showed a heatmap of 1,092 DEGs between high and low stromal scores.

For the immune score, there were 37 upregulated DEGs and 678 downregulated DEGs in the high group compared with the low group. For stromal score, compared with the low score group, there were 160 upregulated DEGs and 932 downregulated DEGs in the high-score group. A Venn diagram showed 18 cross-upregulated DEGs and 435 cross-downregulated DEGs between the immune and stromal groups (Figures 2C,D).

### Function and Pathway Enrichment Analysis of DEGs

Functional enrichment analyses for DEGs, including BP, CC, MF, and KEGG pathways, were conducted using the David gene annotation tool. BP indicated that these genes may be associated with immune response, defense response, response to wounding, inflammatory response, and positive regulation of immune system process. CC indicated that these genes may be associated with intrinsic to membrane, integral to membrane, and plasma membrane. MF indicated that these genes may be associated with carbohydrate binding, cytokine binding, and polysaccharide binding (Figure 2E). The result of KEGG enrichment was related to immune response, including cytokine–cytokine receptor interaction, chemokine signaling pathway, cell adhesion molecules (CAMs), and hematopoietic cell lineage (Figure 2F). Overall, our results confirmed that TME-related DEGs were closely related to the anti-tumor immunity of advanced LUAD patients.

### Construction of Seven-Gene Signature and Survival Analysis

**FIGURE 3:**
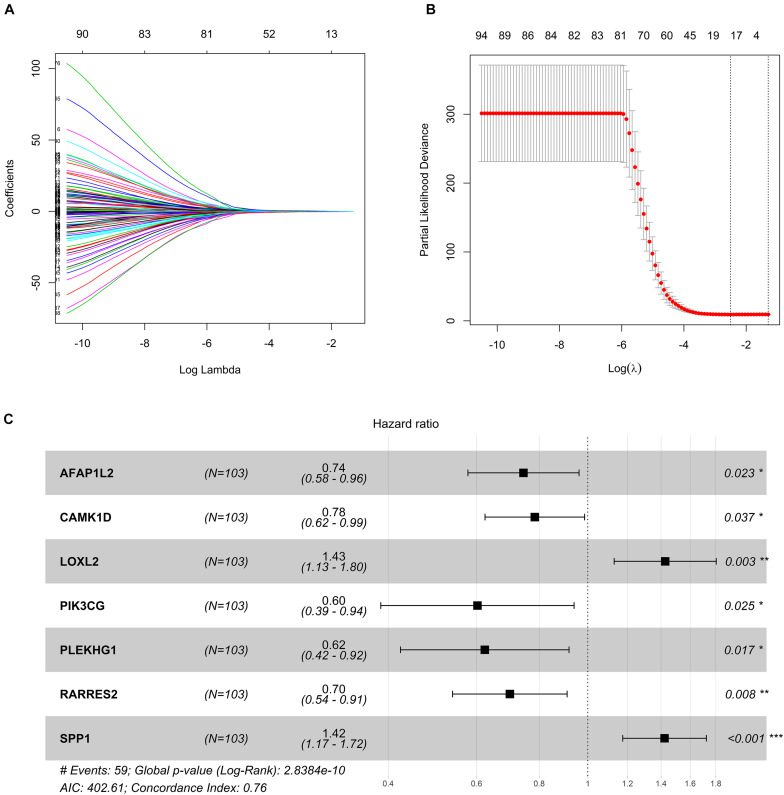
Construction of seven-gene signature. **(A)** LASSO coefficient profiles. **(B)** Tenfold cross-validation result that identified optimal values of the penalty parameter λ. **(C)** Forest plot of seven hub genes based on stepwise regression method and multivariate Cox results. *p < 0.05, **p < 0.01, ***p < 0.001.

In order to explore the potential role of DEGs in survival outcome, a univariate Cox proportional hazards regression model was first conducted, and the results showed that 96 DEGs were selected by univariate analysis. Next, according to the −2 log-likelihood test, the 10-fold cross-validation random sampling method was used, and LASSO regression analysis further screened out 18 DEGs (Figures 3A,B). Finally, a multivariate Cox proportional hazards model was performed, and a total of seven DEGs were selected to establish a seven-gene signature, and the seven-gene signature formula was as follows: risk score = (−0.29529^∗^AFAP1L2) + (−0.24317^∗^CAMK1D) + (0.35563^∗^LOXL2) + (−0.50661^∗^PIK3CG) + (−0.47294^∗^ PLEKHG1) + (−0.35771^∗^ RARRES2) + (0.35258^∗^ SPP1) (Figure 3C).

**FIGURE 4:**
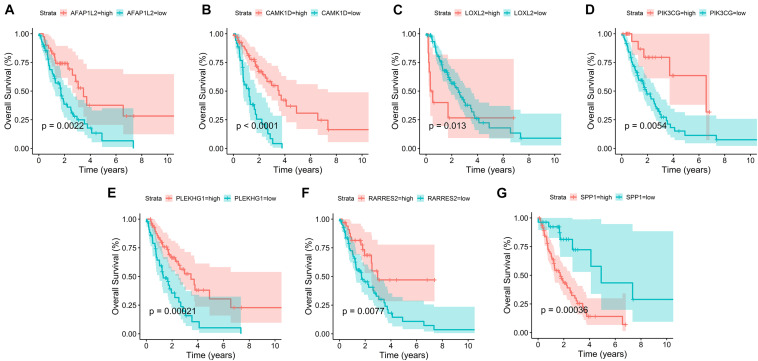
Survival curves of seven prognostic hub genes. **(A**–**G)** Survival curves of seven DEGs were constructed to explore the prognostic value of each gene in the TCGA database.

**FIGURE 5:**
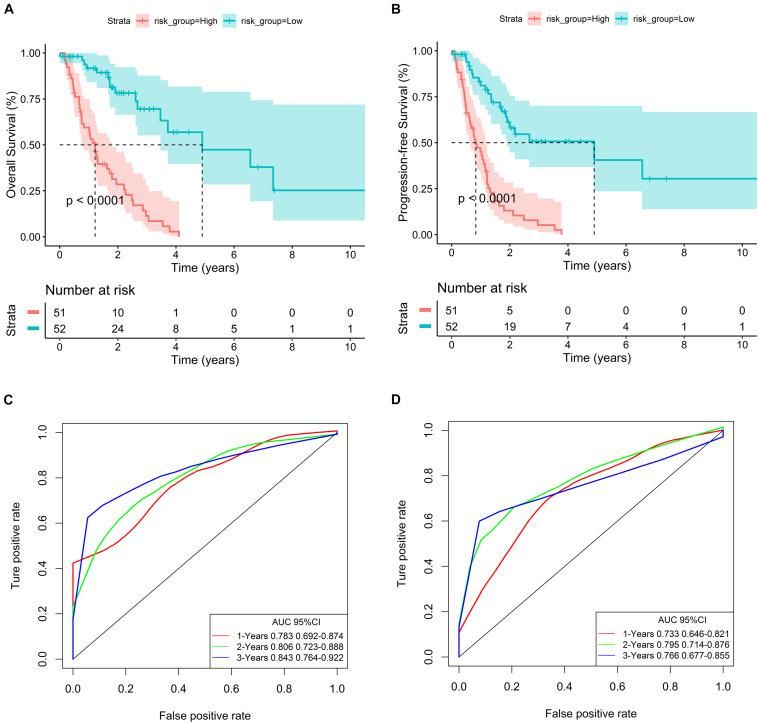
Evaluation of the predictive ability of the seven-gene signature. **(A,B)** K–M curves for OS and PFS of high- and low-risk groups in the TCGA database. **(C,D)** ROC curves for OS and PFS based on the seven-gene signature in the TCGA database.

In addition, survival curves of seven DEGs were constructed to explore the prognostic value of each gene (Figure 4). Furthermore, a total of 51 patients with risk scores higher than the median were classified as “high-risk group,” and the remaining 52 patients were classified as “low-risk group.” K–M curves showed that the OS and PFS of high-risk patients were significantly worse (*p* < 0.001 and *p* < 0.001, respectively; Figures 5A,B).

In order to evaluate the predictive ability of the seven-gene signature, we drew the ROC curve based on the risk score and calculated the AUC of the area under the curve. The AUCs of the first, second, and third year of OS prognostic models were 0.783, 0.806, and 0.843, respectively (Figure 5C). Consistently, the AUCs of the first, second, and third year of PFS prognostic models were 0.733, 0.795, and 0.766, respectively (Figure 5D).

To explore the independent prognostic value of seven-gene signature, multivariate Cox analysis combined with other clinicopathologic characteristics showed that risk score was an independent predictor (For OS, HR: 6.42, 95% CI: 3.32–12.40; For PFS, HR: 4.74, 95% CI: 2.71–8.28) (Table 2).

**TABLE 2:** Multivariate Cox analysis of clinical information and risk group.

Variables	OS		PFS	
	HR (95% CI)	*p*	HR (95% CI)	*p*
Age		0.390		0.331
<65	1		1	
≥65	1.28 (0.73–2.26)		1.30 (0.77–2.19)	
Gender		0.946		0.355
Female	1		1	
Male	1.02 (0.58–1.80)		1.28 (0.76–2.17)	
History of smoking		0.092		0.122
No	1		1	
Yes	2.50 (0.86–7.28)		1.98 (0.83–4.70)	
Tumor laterality		0.719		0.546
Left	1		1	
Right	0.90 (0.49–1.64)		1.19 (0.68–2.09)	
Metastasis		0.205		0.506
M0	1		1	
M1	1.59 (0.78–3.27)		1.25 (0.65–2.39)	
Lymph node status		0.082		0.100
N0	1		1	
N1–N3	2.48 (0.89–6.87)		2.11 (0.87–5.14)	
Pathological T stage		0.360		0.895
T1–T2	1		1	
T3–T4	1.30 (0.74–2.28)		1.04 (0.61–1.76)	
Risk group		<0.001		<0.001
Low	1		1	
High	6.42 (3.32–12.40)		4.74 (2.71–8.28)	

### Validation of the Risk Stratification Model

In the GSE81089 and GSE41271 datasets (Supplementary Figures 1, 2, respectively), the correlation between seven genes and the risk score indicated that AFAP1L2, CAMK1D, PIK3CG, PLEKHG1, and RARRES2 were negatively correlated with the risk score, while LOXL2 and SPP1 were positively correlated with the risk score. The human protein atlas database was used to explore protein expression levels. Typical IHC of four favorable and two adverse prognostic genes (except RARRES2, which was not included in the database) in normal and tumor tissues is shown in Supplementary Figure 3.

**FIGURE 6:**
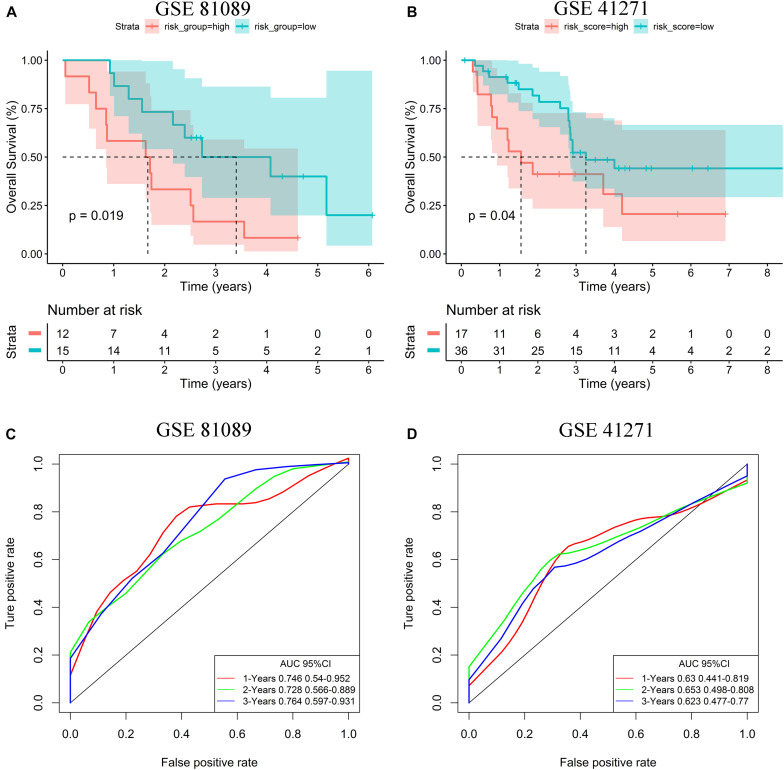
Validation of the risk stratification model. **(A)** K–M curves for OS of high- and low-risk groups in the GSE81089 dataset. **(B)** K–M curves for OS of high- and low-risk groups in the GSE41271 dataset. **(C)** ROC curves for OS based on the seven-gene signature in the GSE81089 dataset. **(D)** ROC curves for OS based on the seven-gene signature in the GSE41271 dataset.

In order to verify the generalization value of the seven-gene signature based on the TCGA cohort, we separately calculated the risk score of each sample for the 27 advanced LUAD patients in GSE81089 and the 53 advanced LUAD patients in GSE41271 using the above risk score formula. For the GSE81089 dataset, K–M survival curves indicated that the low-risk group had higher OS (*p* = 0.019) (Figure 6A). ROC curves based on the risk score model showed that the AUCs for the first, second, and third year of OS prognostic models were 0.746, 0.728, and 0.764, respectively (Figure 6C). Consistently, for the GSE41271 dataset, K–M survival curves indicated that the low-risk group also had higher OS (*p* = 0.04) (Figure 6B). ROC curves based on the risk score model showed that the AUCs for the first, second, and third year of OS prognostic models were 0.630, 0.653, and 0.623, respectively (Figure 6D).

### Estimating the Composition of Immune Cells

**FIGURE 7:**
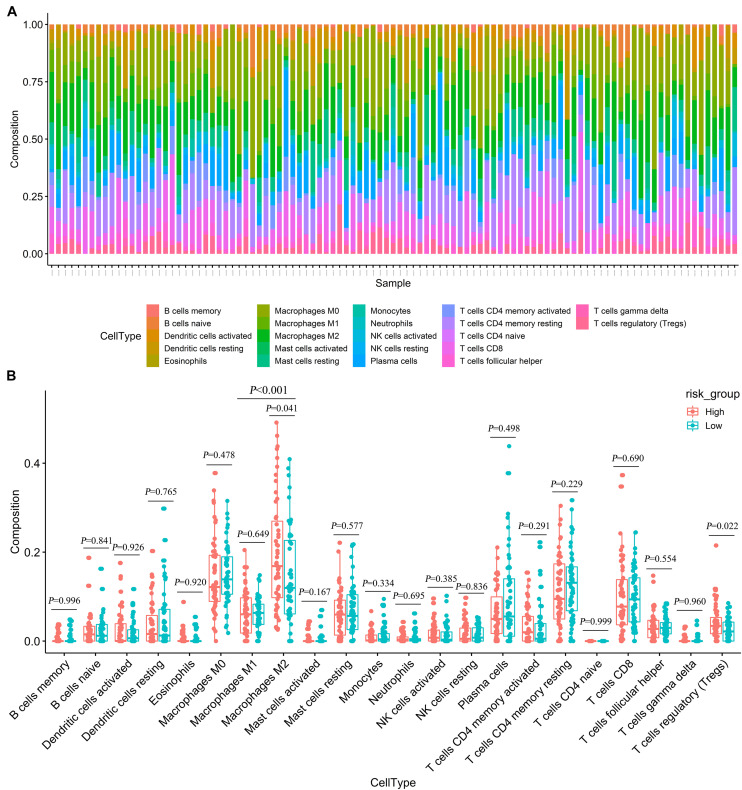
Estimating the composition of immune cells **(A)** Relative proportion of 22 immune cell infiltration in high- and low-risk patients. **(B)** Differences of immune infiltration between high- and low-risk groups.

We used CIBERSORT to estimate the immune cell composition of 103 samples and to quantify the relative levels of different cell types in the mixed cell population (Figure 7A). In patients with advanced LUAD, the expression level of macrophage M2 was significantly higher than that of macrophage M1 (*p* < 0.001). As shown in Figure 7B, we compared different cell types of patients in the low-risk group with those in the high-risk group. These results indicated that the expression levels of macrophages M2 and regulatory T cells (Tregs) in the high-risk group were significantly higher than those in the low-risk group (*p* = 0.041 and *p* = 0.022, respectively).

## Discussion

Early symptoms of LUAD are relatively insidious, without typical symptoms. As a result, most LUAD patients are at an advanced stage at the time of diagnosis, losing the best chance of surgical resection and affecting the treatment effect and quality of life of patients (Shapira, 2018). Fortunately, the treatment of LUAD continues to develop, from the original surgery, radiotherapy, chemotherapy, and targeted therapy to the current tumor immunotherapy, and the continuous innovation of treatment methods provides new treatment options for patients with advanced LUAD (Hanna et al., 2017). Previous studies have shown that TME plays a vital role in malignant progression, immune escape, and therapeutic resistance (Lambrechts et al., 2018). Therefore, it is important to study the TME of advanced LUAD in this study to determine biomarkers that can predict survival outcomes of patients.

In order to study the TME of advanced LUAD, we first calculated the immune score, stromal score, and estimate score of each advanced LUAD sample extracted from the TCGA database by applying an ESTIMATE algorithm. These patients were then divided into high/low immune score groups and high/low stromal score groups, and 453 cross-sectional DEGs were identified.

The GO and KEGG analyses of DEGs showed that DEGs mainly participated in TME, such as immune response, defense response, response to wounding, inflammatory response, and positive regulation of immune system process. These processes may inhibit tumor progression and metastasis, thereby improving the prognosis. We also found that these DEGs have a strong correlation with the immune response and tumor immune microenvironment.

In addition, we applied univariate Cox, LASSO, and multivariate Cox regression model to construct a gene signature based on seven DEGs that were screened from 453 cross-sectional DEGs. According to this gene signature, OS and PFS in the high-risk group were significantly worse than those in the low-risk group. Based on the LASSO model, Ma et al. (2020) established a prognostic model for patients with stage I–IV LUAD (AUC = 0.648). Based on the multivariate Cox model, our prognostic model for patients with advanced LUAD had more powerful predictive ability (The AUCs of the first, second, and third year of OS prognostic models were 0.783, 0.806, and 0.843, respectively). Therefore, survival outcomes in advanced LUAD patients could be well predicted by this seven-gene signature.

Among this seven-gene signature, we found that high expression levels of LOXL2 and SPP1 were associated with poor survival outcomes. In contrast, the higher the expression levels of AFAP1L2, CAMK1D, PIK3CG, PLEKHG1, and RARRES2, the better the survival outcomes. LOXL2 can promote the survival and drug resistance of tumor cells, regulate cell adhesion, movement and invasion, and reshape the TME (Barker et al., 2012). Upregulation of LOXL2 has been shown to promote lung cancer invasion and metastasis (Peng et al., 2017). Peinado et al. (2008) also showed that high LOXL2 expression was associated with reduced survival of patients with NSCLC. SPP1, also known as OPN, is a pleiotropic chemokine involved in the induction of tumor metastasis (Shi and Wang, 2017). In various types of cancer, elevated serum SPP1 levels are frequently detected in patients with metastatic cancer (Chiou et al., 2019). Advanced or metastatic LUAD patients with lower SPP1 levels had significantly superior OS and PFS compared with patients with higher levels (Mack et al., 2008).

Actin filament-associated protein 1-Like 2 (AFAP1L2 also known as XB130) is a novel multifunctional adapter protein (Cho et al., 2019). AFAP1L2 mediates the innate immune response and inhibits tumor lung cancer cell proliferation and metastasis (Wang et al., 2020). CAMK1D, an inhibitory kinase, is a member of the calcium/calmodulin-dependent protein kinase 1 family. CAMK1D overexpression impairs tumor neoangiogenesis *in vivo*, thus achieving tumor inhibition (Dimitrova et al., 2016). PIK3CG is deemed to be a tumor suppressor gene (Kratz et al., 2002). Immunohistochemistry revealed a decreased PIK3CG expression in 85% of colorectal cancers, which was associated with tumor invasiveness and metastasis (Semba et al., 2002). RARRES2 is also known as chemerin (Shin et al., 2018). For LUAD, Yi et al. (Liu-Chittenden et al., 2017) found that the expression level of RARRES2 was positively correlated with NK cells in tumor invasion. Previous studies have also shown that higher RARRES2 expression was associated with positive prognosis in lung cancer patients (Zhao et al., 2011; Cai et al., 2016). PLEKHG1 belongs to a family of Rho-GEFs. Matthew et al. (Traylor et al., 2019) found that genetic variation in PLEKHG1 was associated with white matter hyperintensities and ischemic stroke. However, the relationship between PLEKHG1 and LUAD has not been reported, and PLEKHG1 may be a new therapeutic target for LUAD.

Currently, immunotherapy for advanced LUAD mainly uses checkpoint inhibitors, such as PD-1/PD-L1 inhibitors and CTLA-4 inhibitors, to activate the patient’s own immune system to kill tumor cells. According to the different phenotypes and activation states of macrophages, they are classified into two polarized types: classically activated macrophages (macrophages M1) and alternatively activated macrophages (macrophages M2) (Martinez and Gordon, 2014). The macrophages M2 exhibit immunosuppression, which can promote tumorigenesis, angiogenesis, and metastasis (Noy and Pollard, 2014), and macrophages M1 play a key role in the anti-tumor immune effect (Mantovani et al., 2017). Our results showed that the proportion of macrophages M2 in advanced LUAD patients was significantly higher than that of macrophages M1. Although the ratio of macrophage M1/M2 in the high-risk group was lower than that in the low-risk group, it was not found to be statistically significant in our study, possibly due to the limitation of sample size. For the ratio of macrophage M1/M2, our study can be used as a hint, and further large sample data may be needed to verify this. These may indicate that the late stage of LUAD is related to the differentiation of macrophages M1 into macrophages M2. Interestingly, we also found that for advanced LUAD patients, the expression levels of Tregs and macrophages M2 in the high-risk group were significantly higher than those in the low-risk group. Tregs cells can attenuate the anti-tumor effects of CD4 T, CD8 T, and NK cells (Frydrychowicz et al., 2017). Therefore, combination immunotherapy for inducing macrophages M2 to polarize macrophages M1 and regulating the function of Treg immunosuppressive cells may provide clues for the precise immune treatment of advanced LUAD patients and improving the effect of tumor immunotherapy.

However, this study also had certain limitations. First, this study only conducted bioinformatics research on public databases. Next, we should verify the results of this study through clinical patients in the prospective design. Second, our study provided evidence that seven TME-related genes were significantly related to the prognosis of advanced LUAD patients, but they were analyzed only through data mining merely. The biological function and mechanism of these genes depend on further experimental studies to elucidate.

## Conclusion

In summary, our study established and validated a seven-gene signature associated with TME, which might serve as a prognosis stratification tool to provide a theoretical basis for predicting survival outcomes of advanced LUAD patients. In addition, macrophages M2 polarization may lead to worse prognosis in patients with advanced LUAD.

## Data Availability Statement

The datasets presented in this study can be found in online repositories. The names of the repository/repositories and accession number(s) can be found in the article, further inquiries can be directed to the corresponding author.

## Ethics Statement

Ethics committee approval for our study was not required because the data were obtained from publicly available databases.

## Author Contributions

HZ and CL conceived and designed the experiments. HZ and XZ analyzed the data. HZ and LG wrote the manuscript. SS and CL reviewed the draft. All authors have approved the final manuscript.

## Conflict of Interest

The authors declare that the research was conducted in the absence of any commercial or financial relationships that could be construed as a potential conflict of interest.

## Publisher’s Note

All claims expressed in this article are solely those of the authors and do not necessarily represent those of their affiliated organizations, or those of the publisher, the editors and the reviewers. Any product that may be evaluated in this article, or claim that may be made by its manufacturer, is not guaranteed or endorsed by the publisher.

## Footnotes

1
https://portal.gdc.cancer.gov/

2
http://www.cbioportal.org/

3
https://www.ncbi.nlm.nih.gov/geo/

4
http://david.ncifcrf.gov

5
http://www.proteinatlas.org

## Supplementary Material

Supplementary Figure 1 Correlations between seven gene expression levels and risk score were found in GSE81089 dataset.

Supplementary Figure 2 Correlations between seven gene expression levels and risk score were found in GSE41271 dataset.

Supplementary Figure 3 Typical IHC of seven genes in normal and tumor tissues.
