# Supplementary material for: A Robust Seven-Gene Signature Associated With Tumor Microenvironment to Predict Survival Outcomes of Patients With Stage III–IV Lung Adenocarcinoma
Source: Front Genet. 2021 Sep 6;12:684281. doi: 10.3389/fgene.2021.684281 (PMC8450538; doi:10.3389/fgene.2021.684281)
Supplement: Supplementary Figure 1 — Correlations between seven gene expression levels and risk score were found in GSE81089 dataset. [file Data_Sheet_1.ZIP › Supplement Figure/Supplement Figure Legend.docx]

**Supplement Figure Legend**

**Figure S1.** Correlations between seven gene expression levels and risk score were found in GSE81089 dataset.

**Figure S2.** Correlations between seven gene expression levels and risk score were found in GSE41271 dataset.

**Figure S3.** Typical IHC of seven genes in normal and tumor tissues.
