# Supplementary figures and images for: A Robust Seven-Gene Signature Associated With Tumor Microenvironment to Predict Survival Outcomes of Patients With Stage III–IV Lung Adenocarcinoma
Source: Front Genet. 2021 Sep 6;12:684281. doi: 10.3389/fgene.2021.684281 (PMC8450538; doi:10.3389/fgene.2021.684281)

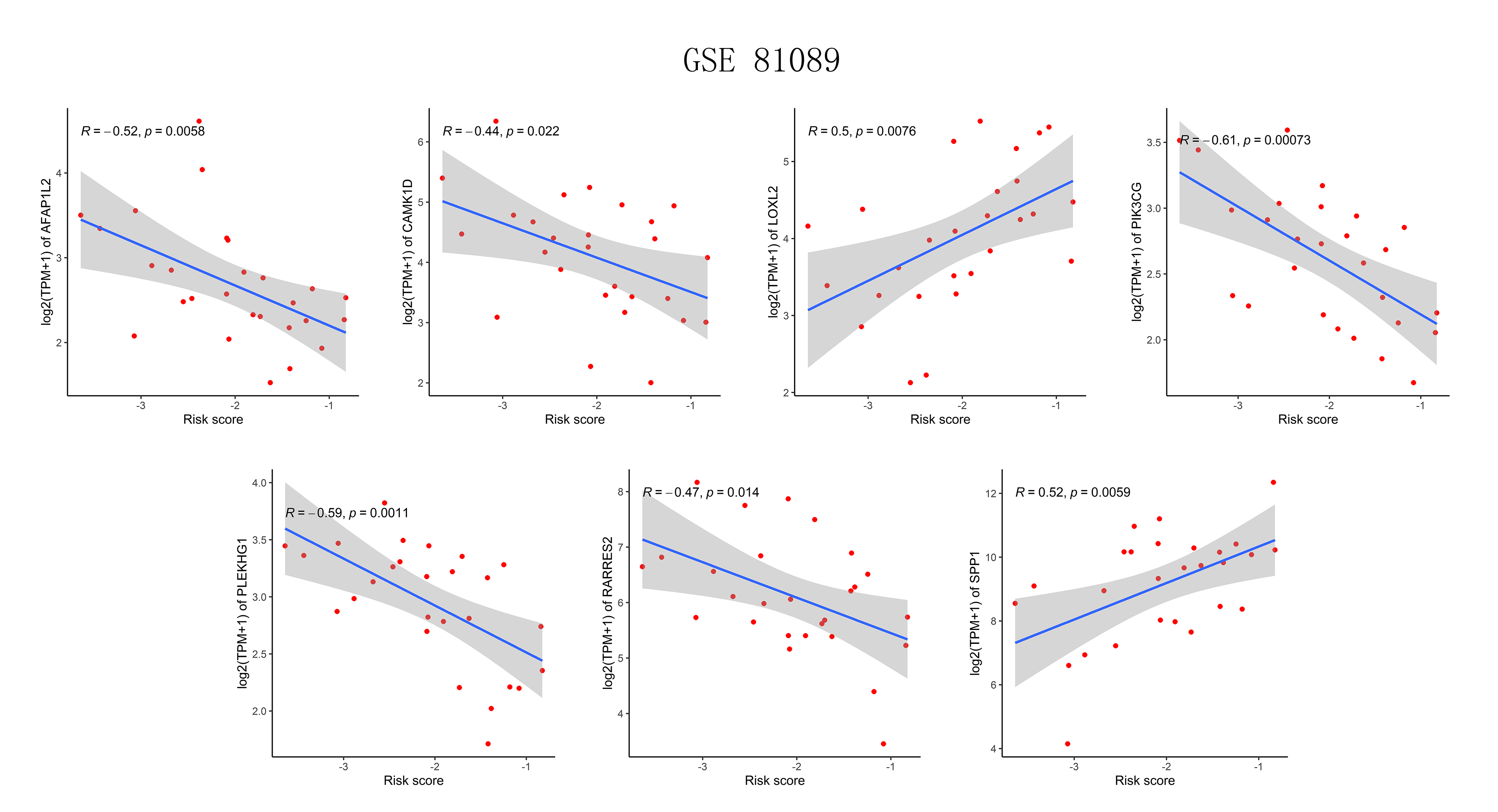

Supplement: Supplementary Figure 1 — Correlations between seven gene expression levels and risk score were found in GSE81089 dataset. [file Data_Sheet_1.ZIP › Supplement Figure/Figure S1.tif]

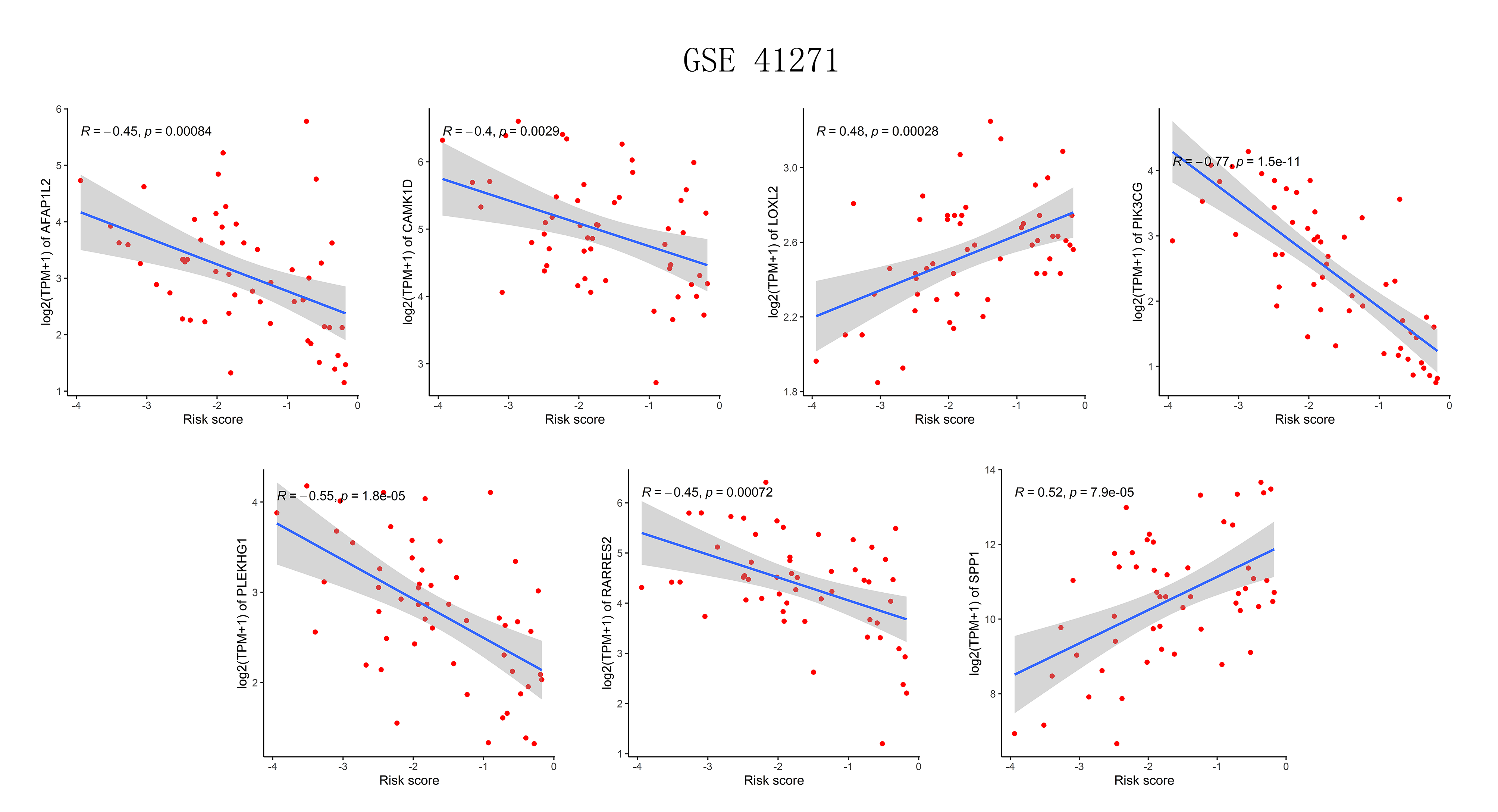

Supplement: Supplementary Figure 1 — Correlations between seven gene expression levels and risk score were found in GSE81089 dataset. [file Data_Sheet_1.ZIP › Supplement Figure/Figure S2.tif]

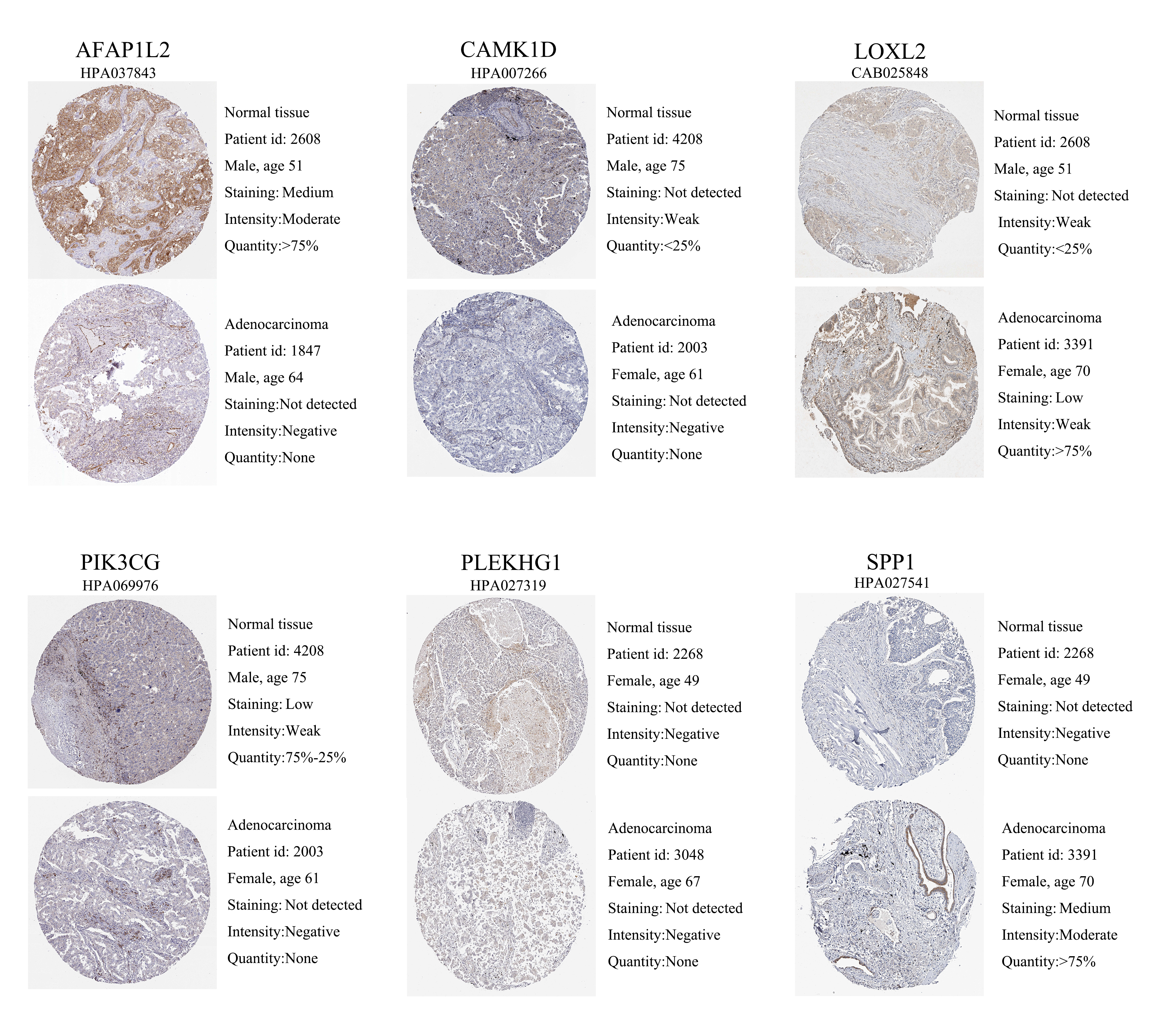

Supplement: Supplementary Figure 1 — Correlations between seven gene expression levels and risk score were found in GSE81089 dataset. [file Data_Sheet_1.ZIP › Supplement Figure/Figure S3.tif]
